# Explaining Health Disparities between Heterosexual and LGB Adolescents by Integrating the Minority Stress and Psychological Mediation Frameworks: Findings from the TRAILS Study

**DOI:** 10.1007/s10964-020-01206-0

**Published:** 2020-02-19

**Authors:** Wouter Kiekens, Chaïm la Roi, Henny M. W. Bos, Tina Kretschmer, Diana D. van Bergen, René Veenstra

**Affiliations:** 1 Department of Sociology/Interuniversity Center for Social Science Theory and Methodology (ICS), University of Groningen, Grote Rozenstraat 31, 9712 TG Groningen, The Netherlands; 2 Swedish Institute for Social Research (SOFI), Stockholm University, Universitetsvägen 10 F, 114 18 Stockholm, Sweden; 3 Institute for Futures Studies, Holländargatan 13, 101 31 Stockholm, Sweden; 4 Department of Education, University of Amsterdam, Nieuwe Achtergracht 127, 1001 NG Amsterdam, The Netherlands; 5 Department of Pedagogy and Educational Science, University of Groningen, Grote Rozenstraat 38, 9712 TJ Groningen, The Netherlands

**Keywords:** Minority stress, Psychological mediation, Substance use, Internalizing problems, Lesbian, gay, bisexual (LGB), Adolescents

## Abstract

Lesbian, gay, and bisexual (LGB) adolescents experience elevated levels of internalizing problems and use more substances than heterosexual adolescents. The minority stress and psychological mediation framework are complementary theoretical frameworks that were developed to explain these disparities. However, limited empirical research has integrated both frameworks to study health disparities between heterosexual and LGB adolescents. This study attempts such an integration, using data from the first five waves (participant age 11–22) of the TRacking Adolescents’ Individual Lives Survey (TRAILS), a cohort study of Dutch adolescents (*N* = 1738; 151 LGB; 54.8% girls). It was tested whether an LGB identity was linked to internalizing problems and substance use through a serial mediation process, in which sexual identity would be associated with peer victimization and negative relationships with parents (first set of mediators, in keeping with the minority stress framework), which in turn would be associated with fear of negative social evaluation and a lack of social support (second set of mediators, in keeping with the psychological mediation framework), and eventually increasing the risk for internalizing problems and elevated levels of substance use. Moreover, it was tested whether the link between minority stress and substance use was mediated by peers’ substance use levels, as hypothesized by the psychological mediation framework. Compared to heterosexual participants, LGB participants reported more internalizing problems, smoked more cigarettes, and used more marijuana, but did not consume more alcohol. The relation between sexual identity and internalizing problems was mediated by peer victimization and parental rejection, which is in line with the minority stress framework. No statistically significant support was found for the psychological mediation framework. These findings provide a better understanding of the pathways through which sexual identity disparities in mental wellbeing and substance use come about.

## Introduction

Adolescents who identify as lesbian, gay, or bisexual (LGB) are at greater risk for developing mental health and substance use problems compared to heterosexual adolescents (Goldbach et al. 2014; Plöderl and Tremblay 2015). Two major theoretical frames are often used for understanding the disproportionate rates of health issues among LGB people. First, the minority stress framework identifies several types of stigma-related stressors that LGB adolescents experience in addition to general stressors (Meyer 2003). These higher rates of (minority) stress among LGB adolescents might explain their higher rates of mental health problems and substance use, where the latter may be seen as a mechanism to cope with minority stressors (Meyer 2003). Second, Hatzenbuehler (2009) extended the minority stress framework by proposing how stigma-related stressors might negatively affect general intra- and interpersonal psychological processes, which, in turn, are related to health and substance use disparities between LGB and heterosexual individuals. This framework has been labeled the psychological mediation framework (Hatzenbuehler 2009).

Both frameworks have been applied to explain differences in mental health and substance use between LGB and heterosexual adolescents (e.g., Baams et al. 2015; Hatzenbuehler et al. 2011; Rosario et al. 2014; Woodford et al. 2012). Unfortunately, however, integrated research is rare, though the combination of both frameworks could provide a better understanding of what drives disparities in mental health and substance use between LGB and heterosexual adolescents. Therefore, the aim of this study was to test the minority stress and the psychological mediation framework in one empirical analysis of health disparities between LGB and heterosexual adolescents.

### Minority Stress Framework

Minority stressors are stigma-related stressors experienced by sexual minority people because of their marginalized sexual identity, in addition to general life stressors (Meyer 2003). Minority stressors exist on a continuum ranging from distal stressors to proximal stressors. Distal stressors comprise external, objective stressful events and conditions. Examples of distal stressors are being rejected by others or being victimized because of one’s sexual identity. Proximal stressors refer to personal perceptions and appraisals of distal stressors by LGB individuals. An example of such a proximal stressor is the application of negative attitudes that exist in society against LGB people to the self, also referred to as internalized homophobia. The experience of these minority stressors by LGB people can lead to poorer mental health compared to heterosexual people (Mongelli et al. 2018) or the use of substances as a maladaptive coping mechanism (Meyer 2003). This article focuses on a number of distal minority stressors including rejection and victimization.

Peers and parents can be sources of minority stress (Russell and Fish 2016). On average, sexual minority adolescents have less positive relationships with peers and parents than heterosexual adolescents, which is linked to differences in mental health between Dutch sexual minority and heterosexual adolescents (Bos et al. 2008). For instance, in the US, homophobic victimization by peers predicted mental health problems in LGB students, especially for girls (Poteat and Espelage 2007). Moreover, sexual minority youth are more often persistently victimized than their heterosexual peers (Robinson et al. 2013) and persistently victimized LGB adolescents reported more internalizing problems (Kaufman et al. 2019). Within the family, parental rejection explained the relation between sexual identity and depressive symptoms in a Dutch sample, especially among lesbian girls and bisexual participants (la Roi et al. 2016). Similarly, the relation between a sexual minority identity and depressive symptoms was partly explained by lower family satisfaction among US adolescents (Luk et al. 2018). Additionally, sexual minority youth reported less closeness and support from parents compared to heterosexual youth, which was linked to lower mental health, especially in US girls compared to boys (Pearson and Wilkinson 2013).

Focusing on substance use, minority stressors such as poorer quality of relationships with peers and parents can account for substance use disparities between heterosexual and LGB youth as well. Victimization by peers in schools, for instance, explained disparities in substance use between LGB and heterosexual adolescents in a representative student sample (Bontempo and D’Augelli 2002). Additionally, LGB college students’ experiences of interpersonal mistreatment explained their higher prevalence of drinking problems compared to heterosexual college students (Woodford et al. 2012). Further, poor mother-child relationship quality explained the association between sexual identity and substance use for LGB emerging adults (Rosario et al. 2014) and parental rejection explained the association between a sexual minority identity and marijuana and hard drug use for women, but not men (Needham and Austin 2010). Similarly, poor parent-child relationship quality explained higher levels of alcohol use of sexual minority youth compared to heterosexual youth, especially for girls (Pearson and Wilkinson 2013). Of note, all studies on substance use have been conducted on US samples.

### Psychological Mediation Framework

Minority stressors such as LGB adolescents’ compromised relationships with peers and parents might explain their elevated risk for mental health problems and substance use. However, this research is limited in that it neglects the role of general intra- and interpersonal psychological processes as intermediate links between minority stressors and mental health and substance use. The psychological mediation framework has been proposed as a refinement of the minority stress framework (Hatzenbuehler 2009). Where the minority stress framework hypothesizes that minority stressors explain links between sexual identity and psychopathology or substance use (Meyer 2003), the psychological mediation framework examines general intra- and interpersonal psychological processes through which minority stressors might affect psychopathology or substance use (Hatzenbuehler 2009). In accordance with the minority stress framework (Meyer 2003) it posits that LGB people are exposed to increased stress resulting from stigma. This stigma-related minority stress is thought to elevate emotion dysregulation, social/interpersonal problems, and cognitive processes that ultimately result in higher risks for psychopathology (Hatzenbuehler 2009). These processes are thought to account for the relation between stigma-related minority stress and psychological problems or substance use. This article focuses on a number of these proposed processes including fear of negative social evaluation, an intrapersonal process, but also social/interpersonal processes such as social support and substance use norms.

Minority stressors such as low relationship quality with peers and parents due to sexual minority status can to a large extent explain associations between sexual identity and health outcomes. Following the psychological mediation framework, it is expected that negative evaluations by others, in turn, mediate the relation between these minority stressors and health outcomes. Although the expected negative evaluations by others are described as a possible consequence of minority stress (Meyer 2003), studies on LGB people hardly examined their role. A recent longitudinal study conducted among emerging adults in the US with concealable stigmatized identities (ranging from being a sexual minority to using drugs) revealed that the expectation to be stigmatized rather than enacted stigma predicted depressive symptoms (Chaudoir and Quinn 2016). More specifically, among US adults, sexual orientation-related rejection sensitivity explained the relation between discrimination and internalizing behaviors (Feinstein et al. 2012). Similarly, chronic expectations of rejection were related to smoking among young sexual minority men in the US (Pachankis et al. 2014) expectations of rejection were related to several internalizing problems among gay and bisexual US university students (Cohen et al. 2016). Thus, for the present study, it was expected that minority stressors such as victimization by peers and cold and unsupportive relationship with parents because of one’s sexual identity would affect one’s mental health and substance use through expected negative evaluations by others.

Social support might also mediate the association between poorer relationship quality with peers and parents, and health outcomes (Hatzenbuehler 2009). It is reasoned that sexual minority people isolate themselves to avoid minority stress experiences such as being rejected (Link et al. 1997). Self-isolation, however, further diminishes their social support, which can affect mental health negatively (Umberson and Karas 2010). In contrast, greater sexual identity-related support by peers and parents has been associated with less emotion-related distress (Doty et al. 2010). Though less studied, support might also work in the opposite direction as relationships with peers tend to be fairly socially and hedonically oriented, sometimes resulting in positive associations between peer support and substance use (Wills et al. 2004).

Permissive substance use norms of peers are a factor that could specifically mediate the association between poorer quality of relationships with peers and parents and substance use. Although empirical support is mixed, it has been argued that minority stressors might contribute to more permissive substance use norms (Hatzenbuehler 2009). Experiences of minority stress might push LGB adolescents into social circles which are characterized by more permissive substance use norms, for example LGB communities with a strong ‘bar culture’. In general, peer’s substance use increases one’s own substance use (Soloski et al. 2016). In fact, sexual minority adolescents’ social networks tend to include more individuals that use substances than those of heterosexual adolescents (Hatzenbuehler et al. 2015). Permissive social norms regarding substance use in one’s network account for the link between sexual identity and alcohol use (Hatzenbuehler et al. 2008). Thus, negative experiences with peers and parents might make a person more vulnerable to permissive substance use norms, resulting in more substance use.

## Current Study

**Fig. 1:**
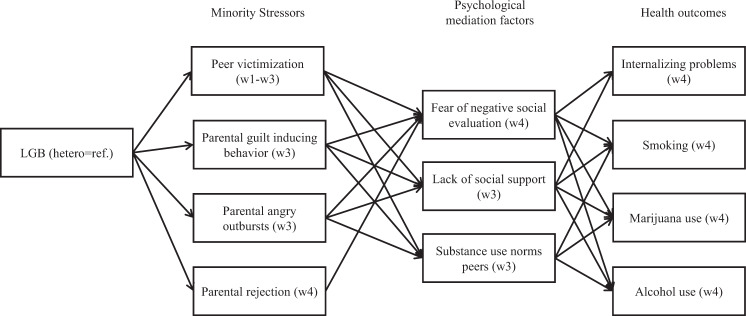
Conceptual model. Note: In statistical models, also effects of sexual orientation on psychological mediation factors and outcomes, and effects of minority stress factors on health outcomes were estimated

The aim of the present study was to explain internalizing problems and substance use disparities between LGB and heterosexual adolescents by focusing on minority stress processes as potential mediators of these disparities, but also included intra- and interpersonal psychological processes that are proposed within the psychological mediation framework to act as mediators of the link between minority stress and health outcomes. It was hypothesized that sexual identity would be associated with peer victimization and negative relationships with parents (first set of mediators, following the minority stress framework), which in turn would be associated with fear of negative social evaluation and lack of social support (second set of mediators, following the psychological mediation framework), which would be positively associated with internalizing problems. A similar serial mediation was expected for substance use. Here, it was hypothesized that sexual identity would be associated with peer victimization and parental rejection (first set of mediators, following the minority stress framework), which would be linked to fear of negative social evaluation, lack of social support, and substance use of peers (second set of mediators, following the psychological mediation framework), which would be positively related to substance use. Figure 1 depicts both hypotheses.

## Methods

### Participants

The data for this study come from the first five waves of the TRacking Adolescents’ Individual Lives Survey (TRAILS), an ongoing prospective cohort study of Dutch youth focused on the development of mental health from childhood to adulthood (Oldehinkel et al. 2015). Children born between 1989 and 1991 were eligible for inclusion in the study. To this end, all primary schools (*N* = 135) in five municipalities, including both rural and urban areas in the North of the Netherlands were approached for participation in the study. Thirteen schools refused participation. Parents or guardians received a personal letter containing information about the study and were contacted by telephone to invite the child and parents or guardians to participate. In total, 210 children were excluded from the study because they were unable to participate or because there was no Dutch-, Turkish-, or-Moroccan speaking parent or guardian available. This yielded a final baseline sample of 2230 children (76% response rate) (*M age* *=* 11.1, 50.8% girls) (De Winter et al. 2005). Participants were followed from pre-adolescence into emerging adulthood. Retention was good with 96.4% at the second wave (*N* *=* 2149, *M age* *=* 13.6, 51.2% girls); 81% at the third wave (*N* *=* 1816, *M age* *=* 16.3, 52% girls); 84% at the fourth wave (*N* *=* 1881, *M age* *=* 19.1, 52% girls); and 80% at the fifth wave (*N* *=* 1778, *M age* *=* 22.3, 53% girls) (Huisman et al. 2008; Oldehinkel et al. 2015). Ethics approval for TRAILS was obtained from the National Dutch Ethics Committee Central Committee on Research Involving Human Subjects (#NL38237.042.11).

### Measures

For each variable the wave of measurement is noted because not all variables were measured at each wave.

### Sexual Identity

Sexual identity was measured using one item that assessed self-identified sexual identity at waves 4 and 5. The question was phrased as follows: “What do you think you are?’ with answer options 1 = *Heterosexual*, 2 = *Homosexual*, and 3 = *Bisexual*. Participants were coded as LGB if they self-identified as homosexual (i.e., lesbian/gay) or bisexual in one or both waves.

### Peer Victimization

Peer victimization was measured using one item from the Youth Self-Report (YSR) (Achenbach and Rescorla 2001) measured at waves 1–3 that read as follows: “I am being bullied a lot”. Answering options were 0 = *Not at all*, 1 = *A little or sometimes*, and 2 = *Clearly or often*. Scores on this variable were highly skewed and preliminary analyses indicated that participants who experienced bullying once already reported higher levels of internalizing problems compared to participants who reported no peer victimization at all. Therefore, peer victimization was recoded into a dummy variable distinguishing between participants never reporting peer victimization and participants experiencing peer victimization *a little or sometimes”* or *clearly or often* at either wave 1, 2, or 3.

### Negative Relationships with Parents

Three constructs were available that measured negative relationships with parents as perceived by adolescents: parental guilt-inducing behaviors, parental angry outbursts, and parental rejection. First, parental guilt-inducing behaviors were operationalized as the mean response to the following statements (assessed for both parents), measured at wave 3: Your father/mother “… avoids you”; “… behaves to you in a silent and cold manner”; “… does not speak to you for long times”. Response options ranged from 0 = *Never* to 4 = (*almost) Always*, and the scale was of adequate internal consistency (*α* *=* 0.77 for guilt-inducing behavior father; *α* = 0.74 for guilt-inducing behavior mother).

Second, parental angry outbursts were operationalized as the mean evaluation of the following items (assessed for both parents), measured at wave 3: Your father/mother “… has angry outburst and tells you off”; “… finds it difficult to hide his/her irritations”; “… argues with you and complains about you loudly”. Response options ranged from 0 = *Never* to 4 = *Almost always*, and the scale was internally consistent (*α* = 0.78 for angry outbursts father; *α* = 0.76 for angry outbursts mother).

Third, parental rejection was measured at wave 4, by means of the EMBU-C (Markus et al. 2003) which includes 4 items for fathers and 4 items for mothers, e.g., “Does your father/mother punish you for minor things?”. Response options ranged from 1 = *No, never* to 4 = *Yes, almost always*. The internal consistency of the scale was moderate (*α* = 0.70 for rejection by the father; *α* = 0.67 for rejection by the mother). Scores on guilt-inducing behavior, angry outbursts, and rejection displayed by fathers and mothers were strongly correlated (*r* rejection = 0.58; *r* angry outbursts *=* 0.55*; r* guilt-inducing behaviors *=* 0.57). Therefore, reports referring to mothers and fathers were combined and the mean response was used. If the participants completed the measure for one parent only, that report was used.

#### Fear of negative social evaluation

At wave 4, fear of negative social evaluation was measured using a four-item scale (e.g., “I always expect criticism”) that reflects a sense of rejection sensitivity (Tops et al. 2008). The mean score was used and answering options ranged from 1 = *Completely false* to 4 = *Completely true*. Cronbach’s alpha was 0.76.

#### Lack of social support

Social support was measured during wave 3 as part of the Event History Calendar (Caspi et al. 1996). Participants were asked how many close friends they had, with a maximum of seven. For each friend, participants were also asked to indicate the extent to which “this friend helped during hard times in the participant’s life”. Response categories were 1 = *Never*, 2 = *Seldom*, 3 = *Sometimes*, 4 = *Often*, and 5 = *Always*. This was reverse coded to reflect a lack of social support. As such, this variable was expected to be associated with sexual identity, minority stress mediators, and outcome variables in the same direction as all other psychological mediation mediators. The sum score of all friends was used. An alternative operationalization based on mean support received did not lead to different results.

#### Substance use norms peers

Substance use norms of peers were operationalized at wave 3 as the proportion of friends of participants that they believed to use substances. Participants indicated on separate items whether 1 = *None* to 4 = *All* of their friends (a) “smoke cigarettes”, (b) “drink alcohol at least weekly”, (c) “get drunk”, or (d) “smoke marijuana”. The mean response on these items was used, which together comprised an internally consistent scale (*α* = 0.80).

#### Internalizing problems

Internalizing problems were measured at wave 4 with the internalizing problem behaviors broadband dimension of the Adult Self Report (ASR) (39 items). The ASR is an evaluation of emotional and behavioral problems in the past six months (Achenbach and Rescorla 2001). Participants were asked to rate the items (e.g., “I worry a lot”, “I refuse to talk”, and “I have difficulties to make and keep friends”) on a 3-point scale (0 = *Not true*, 1 = *A little or sometimes true*, 2 = *Clearly or often true*). Cronbach’s alpha was 0.93.

#### Substance use

Three types of substance use were assessed: cigarette smoking, alcohol use, and marijuana use. Smoking was measured at wave 4, using the following question: “Did you ever smoke cigarettes, even if it was only one cigarette or just a few puffs?”. Response options were 0 = *I have never smoked*, 1 = *I have only smoked once or twice*, 2 = *I used to smoke, but I quit entirely*, 3 = *I smoke every now and then, but not every day*, 4 = *I smoke every day*. Responses were dichotomized to distinguish between participants that never smoked or smoked only once or twice (0–1), and participants who smoke or used to smoke (2–4).

Alcohol use was measured at wave 4 as the number of times participants drank alcohol in the past month. Response options ranged between *0* and *40 times or more*.

Marijuana use was assessed at wave 4 by asking participants whether they had smoked marijuana in the past year. Response options ranged from 0 = *0 times*, to 13 = *40 times or more*. Responses were dichotomized such that they distinguished between participants that had never smoked marijuana (0) and participants who had smoked marijuana within the past year (all other options).

#### Covariates

Gender, age at wave 4, parental socio-economic status and ethnicity were used as control variables. A composite measure of parental socio-economic status was created by adding the *z*-scores of parental occupational status (ISCO-88), parental education and parental income (Veenstra et al. 2005). Ethnicity was operationalized as a dummy (0 = *Ethnic majority*; 1 = *Ethnic minority background*). Participants were coded as having an ethnic minority background when either they or at least one parent was born in a non-Western country.

Similar to a previous TRAILS study (la Roi et al. 2016), it was empirically acknowledged that early childhood adversities might have an impact on the development of mental health later in life. Therefore, the following variables (all parental report) that reflect exposure to early childhood adversities were controlled for: childhood events (e.g., parental divorce, severe illness of one or both parents), parental internalizing problems, and perinatal complications as reported at wave 1. Multivariate analyses furthermore controlled for parents’ wave 2 report of early childhood (age 0–5) stressfulness of life, or long term difficulties (for more details about the instruments: Heininga et al. 2015; la Roi et al. 2016). Lastly, as substance use was among the outcome variables in this study, wave 1 parental past year smoking and alcohol use was controlled for.

### Analytic Strategy

Hypotheses were tested by estimating indirect effects in two serial mediation path models, in Mplus 7.4 (Muthén and Muthén 1998–2012). The first model tested for the presence of mechanisms in line with the minority stress framework. This model estimated whether associations between sexual identity and smoking, marijuana use, alcohol use, and internalizing problems were mediated by peer victimization, parental guilt-inducing behaviors, parental angry outbursts, and parental rejection.

In the second model, the presence of mechanisms in line with the psychological mediation framework was tested, that is, whether associations between minority stressors and substance use and internalizing problems were mediated by fear of negative social evaluation, lack of social support, and substance use norms of peers (only for the minority stress – substance use links). Figure 1 depicts both models. Baseline levels of outcomes were not controlled for in path analyses in order to estimate between-person differences. A discussion of the methodological and conceptual consequences of controlling versus not controlling for prior reports of health outcomes is provided in the section on sensitivity analyses below.

Peer victimization, smoking, and marijuana use were operationalized as dichotomous variables and therefore a robust weighted least squares estimator that employs a diagonal weight matrix was used (ESTIMATOR = WLSMV in Mplus) (e.g., Muthén et al. 2016). Within the path analyses, probit regressions were estimated in models with categorical dependent variables, whereas the continuous latent response variable underlying the observed dichotomous peer victimization variable was used in path coefficients in which peer victimization was an explanatory variable (Muthén et al. 2016). Path models included both dichotomous and scale level variables, Therefore, unstandardized effects with continuous mediators and dependent variables being standardized before estimating path analyses were estimated to optimize the interpretability of path coefficients.

Analyses were performed on all participants for whom information on sexual identity was present (*n* = 1738). In order to prevent loss of cases and potential bias due to missing data in other variables than sexual identity, multiple imputation using chained equations was conducted and 20 imputed datasets created. Predictive mean matching was performed for imputing missing values, using a donor pool of size *k* = 5 for selecting potential donor responses. Predictive mean matching has been shown to be a robust multiple imputation method for imputing non-normal data. Because donor cases are used, plausible values are imputed and the original data distribution is retained (Kleinke 2017; van Buuren 2012; Vink et al. 2014). This was a suitable imputation method for the study variables, because some of them were skewed or had a limited number of response options. Multiple imputations were performed using the *mi impute* functionality in Stata, using Stata version 15.1 (StataCorp 2017). In order to further adjust for non-normality, bootstrapped standard errors on 5000 bootstrap samples were used.

Last, the classical false discovery rate method (FDR) was used to take into account multiple testing (Benjamini and Hochberg 1995). This was done as follows: For both mediation models, an FDR-derived significance threshold (set at 0.05) was used for determining the statistical significance of paths of theoretical interest (all lines drawn in Fig. 1). Furthermore, for each dependent variable, an FDR-derived significance threshold was used for determining the statistical significance of path-specific indirect effects.

## Results

### Descriptive Statistics

Table 1 displays means and standard deviations for the study variables by sexual identity. LGB adolescents reported significantly more peer victimization than heterosexual adolescents. They also reported higher rates of parental angry outbursts and parental rejection than heterosexual adolescents. Further, LGB adolescents reported greater fear of negative social evaluation and internalizing problems. Differences in substance use between LGB and heterosexual adolescents were found as well, with LGB adolescents reporting higher rates of smoking and marijuana use in the past year than heterosexual adolescents. No differences in alcohol use were observed.

**Table 1:** Descriptive statistics of study variables by sexual identity

	Heterosexual (*n* = 1587)	LGB (*n* = 151)	Difference^a^ (LGB-Heterosexual)	95% CI difference
Minority stressors				
Peer victimization (w1–w3)	37%	55%	0.18^**^	[0.09, 0.26]
Parental guilt inducing behavior (w3)	0.27 (0.50)	0.35 (0.62)	0.08	[−0.01, 0.17]
Parental angry outbursts (w3)	1.07 (0.79)	1.25 (0.79)	0.18^**^	[0.05, 0.31]
Parental rejection (w4)	1.45 (0.40)	1.59 (0.54)	0.14^**^	[0.07, 0.22]
Psychological mediators				
Fear of negative social evaluation (w4)	2.35 (0.69)	2.51 (0.77)	0.16^**^	[0.04, 0.27]
Lack of social support (w3)	15.60 (9.08)	15.45 (8.89)	−0.14	[−1.58, 1.29]
Substance use norms peers (w3)	2.52 (0.89)	2.60 (0.93)	0.08	[−0.07, 0.23]
Outcome variables				
Internalizing problems (w4)	0.24 (0.24)	0.37 (0.31)	0.13^**^	[0.08, 0.17]
Smoking (w4)	44%	60%	0.15^**^	[0.07, 0.23]
Marijuana use (w4)	33%	46%	0.13^**^	[0.04, 0.21]
Alcohol use (w4)	6.49 (7.93)	6.21 (8.33)	−0.28	[−1.62, 1.04]
Covariates				
Boy	46%	38%	−0.07	[−0.16, 0.01]
Age (w4)	19.05 (0.58)	19.10 (0.61)	0.05	[−0.05, 0.15]
Parental SES (w1)	0.06 (0.78)	−0.03 (0.77)	−0.09	[−0.22, 0.04]
Ethnic minority (majority = ref.)	8%	11%	0.03	[−0.02, 0.08]
Childhood events (w1)	0.70 (0.87)	0.66 (0.80)	−0.04	[−0.18, 0.11]
Parental internalizing problems (w1)	0.54 (0.80)	0.51 (0.77)	−0.03	[−0.16, 0.10]
Perinatal problems (w1)	1.02 (1.15)	1.03 (1.06)	0.02	[−0.18, 0.21]
Long-term difficulties (w2)	0.51 (0.86)	0.61 (0.94)	0.10	[−0.04, 0.24]
Early life stress (w2)	2.41 (2.03)	2.34 (1.95)	−0.07	[−0.40, 0.26]
Parental smoking (w1)	1.96 (1.33)	2.10 (1.35)	0.14	[−0.08, 0.36]
Parental alcohol use (w1)	2.80 (1.27)	2.83 (1.29)	0.03	[−0.19, 0.23]

Wave of measurement between brackets

^a^F-test on difference in proportions, t-test on difference in means

*p < 0.05; **p < 0.01 two-sided

Table 2 provides correlations. As expected, peer victimization was positively correlated with a lack of social support. Further, all negative patent-child relationship variables were correlated. Parental-guilt inducing behaviors were positively correlated with substance use norms of peers, parental angry outbursts, and parental rejection with fear of negative social evaluation. Furthermore, fear of negative social evaluation was positively correlated with internalizing problems. Last, the substance use norm of peers was positively correlated with own smoking, marijuana use, and alcohol use.

**Table 2:** Pairwise correlations between study variables

	Measure	1	2	3	4	5	6	7	8	9	10	11	12	13	14	15	16	17	18	19	20	21	22	23
1	LGB	.																						
2	Peer victimization (w1–w3)	0.10	.																					
3	Parental guilt inducing behavior (w3)	0.05	0.07	.																				
4	Parental angry outbursts (w3)	0.07	0.05	0.40	.																			
5	Parental rejection (w4)	0.10	0.13	0.26	0.34	.																		
6	Fear of negative social evaluation (w4)	0.07	0.06	0.09	0.16	0.10	.																	
7	Lack of social support (w3)	−0.01	0.12	0.02	−0.04	0.05	0.00	.																
8	Substance use norms peers (w3)	0.03	−0.03	0.15	0.08	0.07	−0.06	−0.09	.															
9	Internalizing problems (w4)	0.14	0.20	0.20	0.27	0.35	0.40	0.07	0.03	.														
10	Smoking (w4)	0.09	−0.00	0.10	0.04	0.13	−0.12	−0.07	0.40	0.07	.													
11	Marijuana use (w4)	0.07	−0.05	0.06	0.07	0.06	−0.00	−0.00	0.28	0.05	0.37	.												
12	Alcohol use (w4)	−0.01	−0.09	−0.01	−0.03	0.02	−0.01	−0.04	0.21	−0.06	0.21	0.22	.											
13	Boy	−0.04	0.03	−0.03	−0.18	−0.06	−0.11	0.22	0.04	−0.20	−0.01	0.15	0.17	.										
14	Age (w4)	0.02	−0.04	0.01	−0.04	−0.03	−0.10	0.03	0.19	−0.00	0.13	0.08	0.08	0.04	.									
15	Parental SES (w1)	−0.03	−0.16	−0.05	0.03	−0.09	0.16	−0.07	−0.07	−0.08	−0.12	0.09	0.15	0.02	−0.06	.								
16	Ethnic minority (majority = ref.)	0.03	−0.03	0.05	0.06	0.10	−0.05	−0.05	0.04	0.06	0.02	0.03	−0.06	−0.05	0.06	−0.13	.							
17	Childhood events (w1)	−0.01	0.11	0.07	0.05	0.11	−0.02	−0.01	0.09	0.16	0.10	0.04	0.00	−0.04	0.08	−0.16	0.01	.						
18	Parental internalizing problems (w1)	−0.01	0.03	0.01	−0.02	0.04	0.03	0.02	0.01	0.11	0.05	0.04	−0.05	−0.02	0.03	−0.08	−0.01	0.35	.					
19	Perinatal problems (w1)	0.00	0.02	0.04	−0.01	0.06	0.06	−0.03	−0.05	0.01	−0.01	−0.03	−0.01	0.03	0.01	−0.03	0.00	0.09	0.17	.				
20	Long-term difficulties (w2)	−0.01	0.07	0.02	0.01	0.05	0.05	0.04	−0.03	0.09	0.02	0.03	−0.02	0.04	0.00	0.01	−0.02	0.23	0.17	0.12	.			
21	Early life stress (w2)	0.03	0.11	0.08	0.07	0.15	−0.00	0.02	0.02	0.19	0.07	0.01	−0.03	−0.01	0.03	−0.10	0.06	0.27	0.16	0.09	0.18	.		
22	Parental smoking (w1)	0.03	0.05	0.02	0.00	0.03	−0.09	0.01	0.17	0.04	0.15	0.07	−0.03	−0.01	0.07	−0.21	−0.01	0.17	0.13	0.01	0.05	0.08	.	
23	Parental alcohol use (w1)	0.01	−0.11	−0.03	−0.03	−0.06	0.06	−0.06	0.07	−0.03	0.03	0.13	0.17	0.01	0.01	0.36	−0.14	−0.06	0.02	−0.02	0.00	−0.06	0.05	.

Correlations > |0.06|: *p* < 0.05

### Serial Mediation Models

#### Minority stress model

**Fig. 2:**
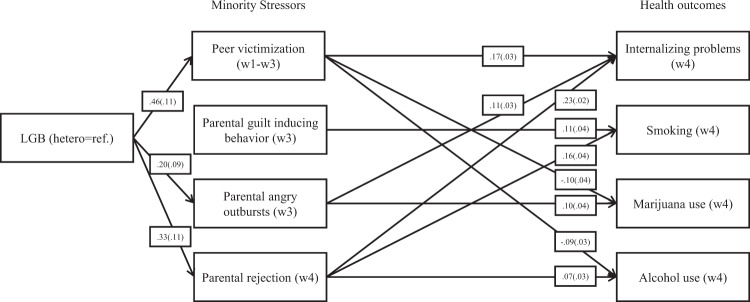
Summary Minority Stress model. Notes: 1. Depicted paths are effects of theoretical interest significant at α = 0.05 after FDR Classical method multiple test correction (Benjamini and Hochberg 1995). Full path model in Appendix A. 2. *N* = 1738, ESTIMATOR = WLSMV, PARAMETERIZATION = THETA, TYPE = IMPUTATION. Unstandardized effects. Bootstrapped standard errors between parentheses. Fully identified model

A fully specified model was estimated, meaning that the number of parameters and variances and covariances were equal in number. Figure 2 shows the paths of theoretical interest that were statistically significant after multiple test correction. Table A1 in online supplementary A contains a complete overview of all model coefficients. As expected, LGB adolescents reported significantly more peer victimization, more angry outbursts by parents, and higher parental rejection compared to heterosexual adolescents. LGB adolescents did not report more guilt-inducing behavior by parents than their heterosexual peers. Significant associations in line with the hypotheses were found between minority stressors and health outcomes as well. Adolescents who reported peer victimization had more internalizing problems than adolescents who did not report peer victimization. Adolescents who reported more guilt-inducing behavior by parents were more likely to smoke. Further, adolescents who reported more angry outbursts by their parents used more marijuana and had more internalizing problems. Moreover, higher rates of parental rejection predicted smoking, alcohol use, and internalizing problems. Contrary to expectations, being victimized was related to a lower likelihood of marijuana use and lower levels of alcohol consumption.

After multiple test correction, two significant path-specific indirect effects in line with the minority stress framework were detected. The association between sexual identity and internalizing problems was mediated both by peer victimization (*b* (*se*) = 0.08 (0.02), 95% *CI* [0.03, 0.12]) and parental rejection (*b* (*se*) = 0.07 (0.02), 95% *CI* [0.03, 0.12]). Furthermore, with all indirect paths between sexual identity and internalizing problems being in the same (positive) direction as the direct effect, the proportion of the association mediated by minority stressors can be calculated by dividing the total indirect effect by the total effect (VanderWeele 2015). Together, minority stress factors mediated 40% of the association between a sexual minority identity and internalizing problems (0.184/0.461).

There were a number of indirect effects that failed to reach statistical significance even though all constituent paths were significant in expected directions. These include the indirect effect of sexual identity on internalizing problems running through parental angry outbursts (*b* (*se*) = 0.02 (0.01), 95% *CI* [0.001, 0.045], not significant after multiple test correction), the indirect effect on marijuana use through parental angry outbursts (*b* (*se*) = 0.02 (0.01), 95% *CI* [−0.004, 0.045]), the indirect effect on smoking through parental rejection (*b* (*se*) = 0.05 (0.02), 95% *CI* [0.009, 0.094], not significant after multiple test correction), and the indirect effect on alcohol use through parental rejection (*b* (*se*) = 0.02 (0.01), 95% *CI* [−0.002, 0.047]).

#### Psychological mediation framework

**Fig. 3:**
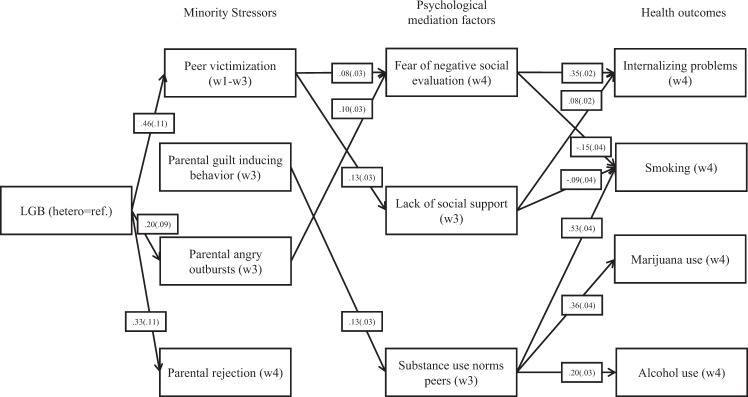
Summary psychological mediation model. Notes: 1. Depicted paths are effects of theoretical interest significant at α = 0.05 after FDR Classical method multiple test correction (Benjamini and Hochberg 1995). Full path model in Appendix A. 2. *N* = 1738, ESTIMATOR = WLSMV, PARAMETERIZATION = THETA, TYPE = IMPUTATION. Unstandardized effects. Bootstrapped standard errors between parentheses. Chi2(3) = 9.028 (no *p*-value provided with MI data); RMSEA = 0.033 (no CI provided with MI data); CFI = 0.998

Next, psychological mediation factors were added to the model. Figure 3 provides a summary of the paths of the psychological mediation model that were statistically significant after multiple test correction. Table A2 in online supplementary A contains all model coefficients. The model fitted the date sufficiently well *χ*^*2*^ = 9.0 (no *p*-value provided with MI data); *RMSEA* = 0.03 (no *CI* provided with MI data); *CFI* = 0.99. As in the previous analyses, LGB adolescents reported more peer victimization, more angry outbursts by parents, and higher parental rejection than heterosexual adolescents. Moving to the link between minority stressors and psychological mediation factors reveals several significant associations in line with theoretical expectations: More peer victimization was related to a lack of social support and greater fear of negative social evaluation. Furthermore, adolescents who reported more guild-inducing behavior by parents had peers with more permissive substance use norms, and adolescents who reported angrier outburst by parents were more afraid of negative social evaluation. With respect to links between psychological mediation factors and health outcomes, Fig. 3 shows several significant effects in line with expectations. Lack of social support and higher levels of fear of negative social evaluation were associated with more internalizing problems. Furthermore, higher substance use norms of peers predicted higher rates of smoking, marijuana use, and alcohol use. Contrary to expectations, however, fear of negative social evaluation was related to a lower likelihood of smoking.

No significant path-specific indirect effects in line with the psychological mediation framework were detected after multiple test correction. Aside from statistical significance, adding psychological mediation factors to the model increased the mediated proportion of the association between sexual identity and internalizing problems. With inclusion of psychological mediation factors, the mediated proportion was 0.52, compared to 0.40 in the minority stress model. Furthermore, there were a number of path-specific indirect effects from sexual identity to internalizing problems that failed to reach statistical significance even though all constituent paths were significant in expected directions. This pertained to the indirect effect of sexual identity on internalizing problems through peer victimization and fear of negative social evaluation (*b* (*se*) = 0.013 (0.006), 95% *CI* [0.001, 0.025], not significant after multiple test correction), the indirect effect through peer victimization and lack of social support (*b* (*se*) = 0.005 (0.002), 95% *CI* [0.000, 0.009], not significant after multiple test correction), and the indirect effect through parental angry outbursts and fear of negative social evaluation (*b* (*se*) = 0.007 (0.004), 95% *CI* [0.000, 0.014]).

### Sensitivity Analyses

Of the participants that provided sexual identity information in both waves 4 and 5, 41 participants identified as heterosexual at wave 4 and as LGB at wave 5, whereas 27 participants identified as LGB at wave 4 yet as heterosexual at wave 5. Two sensitivity analyses were conducted to deal with this discordance. First, analyses omitting participants that identified as heterosexual in one wave, yet as LGB in the other were estimated. This yielded fairly similar results to the ones presented above (available upon request). Only the association between sexual identity and angry parental outburst was somewhat weaker and not statistically significant when using this alternative operationalization of sexual identity. Second, analyses were re-estimated on the full sample, with discordance in sexual identity taken into account by adding a dummy covariate that signaled discordance to the model. After adding this dummy, the association between an LGB identity and parental angry outburst was slightly weaker, whereas the associations between an LGB identity and both peer victimization and parental rejection were slightly stronger compared to the default model. All other path estimates were not noticeably affected, and neither was inference with regard to indirect effects.

Group comparisons to test for gender differences in path coefficients were conducted, as a previous study using TRAILS data (la Roi et al. 2016) showed that sexual identity disparities in depressive symptoms were more pronounced for girls than for boys. The association between sexual identity and both angry outburst and guilt-inducing behavior by parents was stronger for girls compared to boys. Furthermore, the association between guilt-inducing behavior by parents and fear of negative social evaluation was significantly stronger for girls compared to boys.

The default models employed a liberal operationalization of peer victimization, identifying all participants experiencing peer victimization at least once over the course of adolescence, which is in line with research documenting the long-term mental health consequences of bullying victimization (e.g., Takizawa et al. 2014). Results were comparable when using peer victimization measured at wave 3, the latest wave with peer victimization information available. Relatedly, models were re-estimated using alternative dichotomizations of smoking, distinguishing between participants that smoke daily and the rest, and marijuana use, distinguishing between participants who ever used marijuana and the rest. This also did not lead to substantially different results.

Lastly, models were re-estimated controlling for lagged health outcomes in all paths towards health outcomes (lagged dependent variable approach). Lagged dependent variable models were conducted as sensitivity analyses instead of default model specification for both conceptual and statistical reasons: Conceptually, the article aimed to explain differences in adolescent health outcomes *between* LGB and heterosexual adolescents. Consequently, indirect effects should be located on the between-level as well, according to the multilevel mediation literature (Preacher et al. 2010). As the data does not allow for perfectly partialling out within- and between-level variance (most mediators were not measured over time), between-level processes were best approximated by the default models that did not include lagged dependent variables, as including lagged dependent variables would be an attempt to isolate within-person change over time in health outcomes. Moreover, including lagged dependent variables is a suboptimal approach for isolating within-level effects (Hamaker et al. 2015), sometimes leading to less accurate effects estimates than models that do not control for lagged dependent variables (Brüderl and Ludwig 2015; Vaisey and Miles 2017). This being said, re-estimating the path models controlling for lagged dependent variables led to the same overall conclusions, namely that the association between sexual identity and internalizing problems was mediated both by peer victimization and parental rejection, thereby further strengthening confidence in study findings. For an extensive summary of the lagged dependent variable models, please see online supplement B.

## Discussion

Research has repeatedly found that LGB adolescents report more internalizing problems and substance use (smoking, marijuana use, and alcohol use) than their heterosexual peers (Goldbach et al. 2014; Plöderl and Tremblay 2015). The minority stress framework (Meyer 2003) and the psychological mediation framework (Hatzenbuehler 2009) have both been used to explain these disparities between LGB and heterosexual adolescents. However, little research has integrated both frameworks, although doing so provides a more fine-grained understanding of the drivers of disparities in mental health and substance use between LGB and heterosexual adolescents. Therefore, the aim of this study was to examine how indicators of minority stressors (Meyer 2003) and psychological mediators (Hatzenbuehler 2009) together explain disparities between LGB and heterosexual adolescents in internalizing problems and substance use. By integrating both frameworks, health disparities were not only examined by focusing on minority stress processes, but also by taking intra- and interpersonal psychological processes into account that have been proposed as intermediate links between minority stress and health outcomes.

It was hypothesized that sexual identity and internalizing problems would be related through a serial mediation process with peer victimization and negative relationships with parents as the first set of mediators (following the minority stress framework), and fear of negative social evaluation and lower social support as the second set of mediators (following the psychological mediation framework). Similarly, sexual identity and substance use were expected to be linked through peer victimization and negative relationships with parents (first set of mediators, following the minority stress framework) and fear of negative social evaluation, lack of social support, and substance use norms of peers (second set of mediators, following the psychological mediation framework). LGB adolescents reported more internalizing problems, smoked more cigarettes, and consumed more marijuana compared to their heterosexual peers. Mechanisms indicative of minority stressors partially explained these differences. As hypothesized, the association between sexual identity and internalizing problems was mediated by peer victimization and parental rejection. No substantial evidence was found for psychological mediation processes further explaining these health differences between LGB and heterosexual adolescents. Thus, only partial support was found for minority stress processes and no support for psychological mediation processes acting as intermediate links in the sexual identity – minority stress – health outcomes process.

Contrary to expectations, LGB adolescents did not report more alcohol use than their heterosexual peers. This is noteworthy given that a previous meta-analysis found higher alcohol use of LGB adolescents compared to their heterosexual peers (Marshal et al. 2008). Most studies reviewed in this meta-analysis were conducted in the US, which might suggest that sexual identity-based alcohol use disparities are more prevalent there than elsewhere. Alternatively, it might be that only some subgroups of LGB adolescents have a higher risk of alcohol use compared to heterosexual adolescents. Previous research among Dutch adults showed that disparities in substance use between LGB and heterosexual people were driven by the bisexual group (van Beusekom and Kuyper 2018). However, separating the bisexual and lesbian/gay group did not change results. That is, no differences in alcohol use were found when comparing bisexual and heterosexually identified participants (results available upon request). Further, neither peer victimization nor negative parent-child relationship accounted for sexual identity disparities differences in smoking and marijuana use. Although research has established that minority stressors predict substance use of LGB adolescents, not all research has consistently found this pattern for all types of substances. For example, incivility and hostility explained higher rates of LGB students’ drinking problems compared to heterosexual students, but not other drug use (Woodford et al. 2012). Similarly, maternal discomfort with homosexuality did not explain higher rates of smoking among lesbian and gay adolescents (Rosario et al. 2014). Together with this study’s results, these findings imply that using a concept or umbrella term as ‘substance use’ might miss nuances.

This study is not the first to not find strong support for psychological mediation processes (Austin et al. 2004; Wichstrøm and Hegna 2003; Ziyadeh et al. 2007), although prior studies used sexual identity as a proxy for minority stressors. Despite efforts to measure indicators of minority stressors, no support was found for psychological mediation processes. This might reflect that the psychological mediation factors under study are not as important in explaining differences in internalizing problems and substance use among adolescents in the current sample. With regard to more permissive substance use norms of LGB adolescents, it might be that LGB adolescents in the current sample were not able to engage in an ‘LGB bar culture’ because they were either too young to be admitted into these bars, or because no such bars existed in their surroundings (especially in rural areas). With respect to lack of social support, it is feasible that LGB adolescents substitute support. For instance, qualitative research showed that LGB youth more often seek friends online with whom they can talk about their experiences and from whom they receive support (Hillier et al. 2012). Last, focusing on the fear of negative evaluation, the Netherlands is a relatively tolerant country regarding attitudes towards LGB people (van Beusekom and Kuyper 2018), which could result in LGB people expecting or fearing negative evaluation less.

Ultimately, this study aimed to explain internalizing problems and substance use disparities between LGB and heterosexual adolescents by focusing on minority stress processes as potential mediators of these disparities, but also include mediators of the link between minority stress and health outcomes as proposed in the psychological mediation framework. Owing to limitations imposed by secondary data and sample size, only a subset of (distal) minority stress and factors as proposed by the psychological mediation framework were included. Conclusions about the empirical validity of the minority stress and psychological mediation frameworks as a whole are thus beyond the scope of this study. For instance, it could be that that proximal minority stressors or emotion dysregulation and cognitive processes as described in the psychological mediation framework would have been better able to explain health differences between LGB and heterosexual adolescents than the currently employed measures.

Using the TRAILS data enabled us to study internalizing problems and substance use in a general sample of adolescents, but it also came with a drawback. TRAILS was designed as a cohort study on the development of mental health from childhood to adulthood and therefore only general stressors which were used as proxy measures of minority stress were available. For that reason, we cannot be certain to what extent sexual identity disparities in peer victimization and negative parent-child relationships actually reflect minority stress processes. Past studies have made similar assumptions when using general measures for minority stress processes (Katz-Wise and Hyde 2012); these studies are thus comparable to the present work. What is more, LGB participants reported more peer victimization, parental angry outbursts, and parental rejection than heterosexual participants even after controlling for a long list of potentially confounding factors, which affirms that LGB adolescents experience additional (minority) stress in peer and parent-child relationships compared to their heterosexual counterparts.

Related to this, TRAILS included proxy measures of distal minority stressors such as rejection and victimization, but no measures of proximal stressors such as internalized homophobia. Therefore, the present study was unable to study all components proposed in the minority stress framework. Future studies that focus specifically on LGB adolescents should aim to measure minority stress more precise and complete.

In addition, the role of negative relationships with parents as minority stressors was assessed using three measures, guilt inducing behavior, parental angry outbursts, and parental rejection. To the best of our knowledge, no earlier studies on this topic looked at the former two of these or closely related constructs, as most existing work focused on either parental rejection or support (Bouris et al. 2010; Russell and Fish 2016). Although all three indicators of negative parent-child relationship were associated with sexual identity and health outcomes in expected directions, associations were stronger for parental rejection and angry outbursts than guilt-inducing behaviors. A tentative conclusion would thus be that experiencing parental rejection or anger are more important emotional dimensions of the family context than guilt-inducing parental behavior for explaining sexual identity disparities in health outcomes.

Further, although using data from a longitudinal study, we were not able to disentangle how minority stress mediators influenced changes in psychological mediation processes and how both influenced changes in the health outcomes over time. In order to estimate whether a causal relation exists between two variables, a study design is needed that is able to prevent bias in the estimates due to reverse causation, incorrect specification of the lag of the effect, and confounding. Methods that can take all these issues into account require at least three measurements of both dependent and independent variables (Allison et al. 2017; Hamaker et al. 2015; Leszczensky and Wolbring 2019), which were not available in TRAILS. Future research that has at least three measures of all variables at study would be able to overcome these issues with estimating how characteristics induce change in one another.

Lastly, although no significant evidence for indirect effects in line with the psychological mediation framework was found, several of the path estimates in the integrated mediation model were in line with expectations (Fig. 3/Table A2 in online supplementary A). For instance, an LGB identity was related to more peer victimization and parental angry outbursts, which were associated with fear of negative social evaluation and lack of social support, which, in turn, were associated with higher levels of internalizing problem behaviors. The indirect effects running through these paths did not reach statistical significance, however, which might be a consequence of insufficient statistical power to detect small effects.

## Conclusion

This study integrated the minority stress framework and the psychological mediation framework and tested them simultaneously in a Dutch cohort sample of adolescents. In line with the minority stress framework, we found that the relation between sexual identity and internalizing problems was mediated by peer victimization as well as by parental rejection. No significant indirect effects in line with the psychological mediation framework were found. By integrating both frameworks, a more fine-grained understanding of disparities in internalizing problems and substance use between LGB and heterosexual adolescents was achieved. Of note, the differences in internalizing problems, smoking, and marijuana use in this Dutch sample indicate that even in a country known to be relatively LGB-friendly (van Beusekom and Kuyper 2018), LGB adolescents experience health disparities relative to their heterosexual peers. This underlines that further societal acceptance is needed to prevent the setback position that LGB people currently have.

## Supplementary Information

Appendix A

Appendix B
