# Supplementary material for: Explaining Health Disparities between Heterosexual and LGB Adolescents by Integrating the Minority Stress and Psychological Mediation Frameworks: Findings from the TRAILS Study
Source: J Youth Adolesc. 2020 Feb 19;49(9):1767–82. doi: 10.1007/s10964-020-01206-0 (PMC7423798; doi:10.1007/s10964-020-01206-0)
Supplement: Supplementary file 1 — Appendix A [file 10964_2020_1206_MOESM1_ESM.docx]

**Online Supplementary A: full path model**

| Table A1  *Model Coefficients Minority Stress Model* | | |  |
| --- | --- | --- | --- |
| Path coefficients | b | SE | 95% CI |
| *Peer victimization* |  |  |  |
| LGB (Heterosexual=ref.) | 0.46 | 0.11 *^**^* | [0.24, 0.68] |
| Boy (girl=ref.) | 0.09 | 0.06 | [-0.03, 0.22] |
| Age wave 4 | -0.11 | 0.06 *^*^* | [-0.22, -0.01] |
| Parental SES | -0.22 | 0.05 *^**^* | [-0.31, -0.13] |
| Ethnic minority (majority=ref.) | -0.31 | 0.12 *^*^* | [-0.55, -0.07] |
| Childhood events | 0.10 | 0.04 *^*^* | [0.02, 0.18] |
| Parental internalizing problems | -0.03 | 0.04 | [-0.11, 0.06] |
| Perinatal problems | 0.00 | 0.03 | [-0.05, 0.06] |
| Long-term difficulties | 0.11 | 0.04 *^*^* | [0.03, 0.19] |
| Early life stress | 0.03 | 0.02 | [-0.00, 0.07] |
| Parental smoking | 0.01 | 0.03 | [-0.04, 0.06] |
| Parental alcohol use | -0.07 | 0.03 ^*^ | [-0.13, -0.01] |
|  |  |  |  |
| *Guilt inducing parents* |  |  |  |
| LGB (Heterosexual=ref.) | 0.15 | 0.11 | [-0.06, 0.36] |
| Boy (girl=ref.) | -0.05 | 0.05 | [-0.15, 0.06] |
| Age wave 4 | 0.01 | 0.05 | [-0.08, 0.09] |
| Parental SES | -0.03 | 0.04 | [-0.11, 0.05] |
| Ethnic minority (majority=ref.) | 0.13 | 0.11 | [-0.09, 0.35] |
| Childhood events | 0.07 | 0.04 | [0.00, 0.14] |
| Parental internalizing problems | -0.04 | 0.04 | [-0.11, 0.03] |
| Perinatal problems | 0.03 | 0.02 | [-0.02, 0.07] |
| Long-term difficulties | 0.07 | 0.04 | [-0.01, 0.15] |
| Early life stress | 0.00 | 0.02 | [-0.03, 0.04] |
| Parental smoking | 0.00 | 0.02 | [-0.04, 0.05] |
| Parental alcohol use | -0.01 | 0.02 | [-0.05, 0.04] |
|  |  |  |  |
| *Angry outbursts parents* |  |  |  |
| LGB (Heterosexual=ref.) | 0.20 | 0.09 *^*^* | [0.04, 0.37] |
| Boy (girl=ref.) | -0.35 | 0.05 *^**^* | [-0.45, -0.25] |
| Age wave 4 | -0.07 | 0.05 | [-0.16, 0.02] |
| Parental SES | 0.06 | 0.04 | [-0.02, 0.14] |
| Ethnic minority (majority=ref.) | 0.20 | 0.11 | [-0.02, 0.43] |
| Childhood events | 0.06 | 0.03 | [-0.01, 0.12] |
| Parental internalizing problems | -0.05 | 0.04 | [-0.12, 0.02] |
| Perinatal problems | 0.00 | 0.02 | [-0.04, 0.04] |
| Long-term difficulties | 0.07 | 0.03 *^*^* | [0.00, 0.14] |
| Early life stress | 0.00 | 0.02 | [-0.03, 0.03] |
| Parental smoking | 0.00 | 0.02 | [-0.04, 0.05] |
| Parental alcohol use | 0.01 | 0.02 | [-0.04, 0.05] |
|  |  |  |  |
| *Parental rejection* |  |  |  |
| LGB (Heterosexual=ref.) | 0.33 | 0.11 *^**^* | [0.12, 0.54] |
| Boy (girl=ref.) | -0.09 | 0.05 | [-0.19, 0.00] |
| Age wave 4 | -0.09 | 0.04 | [-0.17, 0.00] |
| Parental SES | -0.06 | 0.04 | [-0.13, 0.01] |
| Ethnic minority (majority=ref.) | 0.29 | 0.11 *^**^* | [0.08, 0.49] |
| Childhood events | 0.08 | 0.03 *^*^* | [0.01, 0.14] |
| Parental internalizing problems | -0.01 | 0.04 | [-0.08, 0.06] |
| Perinatal problems | 0.03 | 0.02 | [-0.01, 0.08] |
| Long-term difficulties | 0.14 | 0.04 *^**^* | [0.07, 0.21] |
| Early life stress | 0.01 | 0.02 | [-0.02, 0.04 |
| Parental smoking | 0.00 | 0.02 | [-0.04, 0.04] |
| Parental alcohol use | -0.02 | 0.02 | [-0.07, 0.03] |
|  |  |  |  |
| *Internalizing problems* |  |  |  |
| LGB (Heterosexual=ref.) | 0.28 | 0.10 *^**^* | [0.09, 0.47] |
| Boy (girl=ref.) | -0.33 | 0.04 *^**^* | [-0.42, -0.25] |
| Age wave 4 | 0.02 | 0.04 | [-0.06, 0.10] |
| Parental SES | 0.00 | 0.03 | [-0.07, 0.06] |
| Ethnic minority (majority=ref.) | 0.09 | 0.09 | [-0.08, 0.27] |
| Childhood events | 0.06 | 0.03 | [-0.01, 0.13] |
| Parental internalizing problems | 0.08 | 0.03 *^*^* | [0.02, 0.15] |
| Perinatal problems | -0.03 | 0.02 | [-0.07, 0.01] |
| Long-term difficulties | 0.11 | 0.04 *^**^* | [0.03, 0.18] |
| Early life stress | 0.02 | 0.02 | [-0.01, 0.04] |
| Parental smoking | -0.01 | 0.02 | [-0.05, 0.03] |
| Parental alcohol use | 0.02 | 0.02 | [-0.02, 0.06] |
| Peer victimization | 0.17 | 0.03 *^**^* | [0.11, 0.23] |
| Parental guilt inducing | 0.06 | 0.03 *^*^* | [0.00, 0.12] |
| Parental angry outbursts | 0.11 | 0.03 *^**^* | [0.06, 0.17] |
| Parental rejection | 0.23 | 0.03 *^**^* | [0.18, 0.28] |
|  |  |  |  |
| *Smoking* |  |  |  |
| LGB (Heterosexual=ref.) | 0.34 | 0.11 *^**^* | [0.11, 0.56] |
| Boy (girl=ref.) | 0.00 | 0.07 | [-0.13, 0.13] |
| Age wave 4 | 0.24 | 0.06 *^**^* | [0.13, 0.35] |
| Parental SES | -0.18 | 0.05 *^**^* | [-0.27, -0.09] |
| Ethnic minority (majority=ref.) | -0.03 | 0.12 | [-0.27, 0.21] |
| Childhood events | 0.07 | 0.04 | [-0.01, 0.16] |
| Parental internalizing problems | 0.00 | 0.04 | [-0.08, 0.09] |
| Perinatal problems | -0.03 | 0.03 | [-0.09, 0.03] |
| Long-term difficulties | 0.04 | 0.04 | [-0.05, 0.13] |
| Early life stress | 0.00 | 0.02 | [-0.04, 0.04] |
| Parental smoking | 0.10 | 0.03 ^**^ | [0.05, 0.15] |
| Parental alcohol use | 0.07 | 0.03 ^*^ | [0.02, 0.13] |
| Peer victimization | -0.08 | 0.04 | [-0.16, 0.00] |
| Parental guilt inducing | 0.11 | 0.04 *^**^* | [0.03, 0.18] |
| Parental angry outbursts | -0.04 | 0.04 | [-0.12, 0.03] |
| Parental rejection | 0.16 | 0.04 *^**^* | [0.09, 0.23] |
|  |  |  |  |
| *Marijuana use* |  |  |  |
| LGB (Heterosexual=ref.) | 0.37 | 0.12 *^**^* | [0.14, 0.60] |
| Boy (girl=ref.) | 0.46 | 0.07 *^**^* | [0.32, 0.59] |
| Age wave 4 | 0.14 | 0.06 *^*^* | [0.02, 0.25] |
| Parental SES | 0.13 | 0.05 *^*^* | [0.03, 0.23] |
| Ethnic minority (majority=ref.) | 0.21 | 0.13 | [-0.04, 0.46] |
| Childhood events | 0.06 | 0.05 | [-0.03, 0.14] |
| Parental internalizing problems | 0.05 | 0.05 | [-0.04, 0.14] |
| Perinatal problems | -0.05 | 0.03 | [-0.11, 0.01] |
| Long-term difficulties | 0.00 | 0.05 | [-0.09, 0.09] |
| Early life stress | 0.01 | 0.02 | [-0.03, 0.04] |
| Parental smoking | 0.08 | 0.03 ^**^ | [0.02, 0.13] |
| Parental alcohol use | 0.11 | 0.03 ^**^ | [0.05, 0.16] |
| Peer victimization | -0.10 | 0.04 *^*^* | [-0.19, -0.02] |
| Parental guilt inducing | 0.04 | 0.04 | [-0.03, 0.12] |
| Parental angry outbursts | 0.10 | 0.04 *^*^* | [0.02, 0.18] |
| Parental rejection | 0.06 | 0.04 | [-0.02, 0.13] |
|  |  |  |  |
| *Alcohol use* |  |  |  |
| LGB (Heterosexual=ref.) | 0.02 | 0.09 | [-0.15, 0.19] |
| Boy (girl=ref.) | 0.35 | 0.05 *^**^* | [0.25, 0.45] |
| Age wave 4 | 0.13 | 0.04 *^**^* | [0.05, 0.21] |
| Parental SES | 0.12 | 0.03 *^**^* | [0.05, 0.18] |
| Ethnic minority (majority=ref.) | -0.15 | 0.10 | [-0.34, 0.04] |
| Childhood events | 0.07 | 0.03 ^*^ | [0.01, 0.14] |
| Parental internalizing problems | -0.07 | 0.03 ^*^ | [-0.13, -0.01] |
| Perinatal problems | 0.00 | 0.02 | [-0.04, 0.03] |
| Long-term difficulties | -0.01 | 0.03 | [-0.08, 0.05] |
| Early life stress | -0.01 | 0.01 | [-0.04, 0.01] |
| Parental smoking | -0.01 | 0.02 | [-0.05, 0.02] |
| Parental alcohol use | 0.10 | 0.02 ^**^ | [0.06, 0.14] |
| Peer victimization | -0.09 | 0.03 *^**^* | [-0.15, -0.03] |
| Parental guilt inducing | -0.01 | 0.03 | [-0.06, 0.05] |
| Parental angry outbursts | -0.01 | 0.03 | [-0.07, 0.05] |
| Parental rejection | -0.07 | 0.03 *^*^* | [0.02, 0.12] |
|  |  |  |  |
| *Peer victimization covariance with* |  |  |  |
| Parental guilt inducing | 0.07 | 0.03 | [0.00, 0.13] |
| Parental angry outbursts | 0.06 | 0.03 | [0.00, 0.12] |
| Parental rejection | 0.12 | 0.03*^**^* | [0.06, 0.18] |
|  |  |  |  |
| *Parental guilt inducing covariance with* |  |  |  |
| Parental angry outbursts | 0.39 | 0.03 *^**^* | [0.30, 0.45] |
| Parental rejection | 0.23 | 0.03 *^**^* | [0.17, 0.29] |
|  |  |  |  |
| *Parental angry outbursts covariance with* |  |  |  |
| Parental rejection | 0.31 | 0.03*^**^* | [0.25, 0.36] |
|  |  |  |  |
| *Internalizing problems covariance with* |  |  |  |
| Smoking | 0.00 | 0.03 | [-0.05, 0.06] |
| Marijuana use | 0.07 | 0.03 ^*^ | [0.01, 0.12] |
| Alcohol use | -0.01 | 0.02 | [-0.04, 0.03] |
|  |  |  |  |
| *Smoking covariance with* |  |  |  |
| Marijuana use | 0.57 | 0.03*^**^* | [0.51, 0.63] |
| Alcohol use | 0.27 | 0.03 *^**^* | [0.20, 0.34] |
|  |  |  |  |
| *Marijuana use covariance with* |  |  |  |
| Alcohol use | 0.20 | 0.03 *^**^* | [0.15, 0.26] |
|  |  |  |  |
| *Intercepts* |  |  |  |
| Parental guilt inducing | 0.00 | 0.04 | [-0.07, 0.07] |
| Parental angry outbursts | 0.12 | 0.04 ^**^ | [0.05, 0.20] |
| Parental rejection | -0.01 | 0.03 | [-0.08, 0.06] |
| Internalizing problems | 0.11 | 0.03 ^**^ | [0.05, 0.17] |
| Alcohol use | -0.14 | 0.03 *^**^* | [-0.20, -0.08] |
|  |  |  |  |
| *Thresholds* |  |  |  |
| Peer victimization$1 | 0.36 | 0.05 ^**^ | [0.27, 0.45] |
| Smoking $1 | 0.14 | 0.05 *^**^* | [0.04, 0.21] |
| Marijuana use$1 | 0.68 | 0.05 *^**^* | [0.58, 0.77] |
|  |  |  |  |
| *Residual variances* |  |  |  |
| Parental guilt inducing | 0.98 | 0.07 *^**^* | [0.85, 1.12] |
| Parental angry outbursts | 0.95 | 0.03 *^**^* | [0.89, 1.01] |
| Parental rejection | 0.94 | 0.04 *^**^* | [0.86, 1.03] |
| Internalizing problems | 0.75 | 0.04 *^**^* | [0.68, 0.82] |
| Alcohol use | 0.91 | 0.06 *^**^* | [0.79, 1.03] |
| Note: ^*^ *p* < 0.05, ^**^ *p* < 0.01, two-sided. | | |  |

| Table A2  *Model Coefficients Psychological Mediation Model* | | |  |
| --- | --- | --- | --- |
| Path coefficients | b | SE | 95% CI |
| *Peer victimization* |  |  |  |
| LGB (Heterosexual=ref.) | 0.46 | 0.11*^**^* | [0.24, 0.68] |
| Boy (girl=ref.) | 0.09 | 0.06 | [-0.03, 0.22] |
| Age wave 4 | -0.11 | 0.06 *^*^* | [-0.22, -0.01] |
| Parental SES | -0.22 | 0.05 *^**^* | [-0.31, -0.13] |
| Ethnic minority (majority=ref.) | -0.31 | 0.12 *^**^* | [-0.55, -0.07] |
| Childhood events | 0.10 | 0.04 *^*^* | [0.02, 0.18] |
| Parental internalizing problems | -0.03 | 0.04 | [-0.11, 0.06] |
| Perinatal problems | 0.00 | 0.03 | [-0.05, 0.06] |
| Long-term difficulties | 0.11 | 0.04 *^*^* | [0.03, 0.19] |
| Early life stress | 0.03 | 0.02 | [0.00, 0.07] |
| Parental smoking | 0.01 | 0.03 | [-0.04, 0.06] |
| Parental alcohol use | -0.07 | 0.03 ^*^ | [-0.13, -0.01] |
|  |  |  |  |
| *Guilt inducing parents* |  |  |  |
| LGB (Heterosexual=ref.) | 0.15 | 0.11 | [-0.06, 0.36] |
| Boy (girl=ref.) | -0.05 | 0.05 | [-0.15, 0.06] |
| Age wave 4 | 0.01 | 0.05 | [-0.08, 0.09] |
| Parental SES | -0.04 | 0.04 | [-0.11, 0.05] |
| Ethnic minority (majority=ref.) | 0.13 | 0.11 | [-0.09, 0.35] |
| Childhood events | 0.07 | 0.04 | [0.00, 0.14] |
| Parental internalizing problems | -0.04 | 0.04 | [-0.11, 0.03] |
| Perinatal problems | 0.03 | 0.03 | [-0.02, 0.07] |
| Long-term difficulties | 0.07 | 0.04 | [-0.01, 0.15] |
| Early life stress | 0.02 | 0.02 | [-0.03, 0.04] |
| Parental smoking | 0.00 | 0.02 | [-0.04, 0.05] |
| Parental alcohol use | -0.01 | 0.02 | [-0.05, 0.04] |
|  |  |  |  |
| *Angry outbursts parents wave 3* |  |  |  |
| LGB (Heterosexual=ref.) | 0.20 | 0.09 *^*^* | [0.04, 0.37] |
| Boy (girl=ref.) | -0.35 | 0.05 *^**^* | [-0.45, 0.25] |
| Age wave 4 | -0.07 | 0.05 | [-0.16, 0.02] |
| Parental SES | 0.06 | 0.04 | [-0.02, 0.14] |
| Ethnic minority (majority=ref.) | 0.20 | 0.11 | [-0.02, 0.43] |
| Childhood events | 0.06 | 0.03 | [-0.01, 0.12] |
| Perinatal problems | 0.00 | 0.02 | [-0.04, 0.04] |
| Long-term difficulties | 0.07 | 0.03 *^*^* | [0.00, 0.14] |
| Parental internalizing problems | -0.05 | 0.04 | [-0.12, 0.02] |
| Early life stress | 0.00 | 0.02 | [-0.03, 0.03] |
| Parental smoking | 0.00 | 0.02 | [-0.04, 0.05] |
| Parental alcohol use | 0.01 | 0.02 | [-0.04, 0.05] |
|  |  |  |  |
| *Parental rejection wave 4* |  |  |  |
| LGB (Heterosexual=ref.) | 0.33 | 0.11 *^**^* | [0.12, 0.54] |
| Boy (girl=ref.) | -0.09 | 0.05 | [-0.19, 0.00] |
| Age wave 4 | -0.09 | 0.04 | [-0.17, 0.00] |
| Parental SES | -0.06 | 0.04 | [-0.13, 0.01] |
| Ethnic minority (majority=ref.) | 0.29 | 0.11 *^**^* | [0.08, 0.49] |
| Childhood events | 0.08 | 0.03 *^*^* | [0.01, 0.14] |
| Perinatal problems | 0.03 | 0.02 | [-0.01, 0.08] |
| Long-term difficulties | 0.14 | 0.04 *^**^* | [0.07, 0.21] |
| Parental internalizing problems | -0.01 | 0.04 | [-0.08, 0.06] |
| Early life stress | 0.01 | 0.02 | [-0.02, 0.04] |
| Parental smoking | 0.00 | 0.02 | [-0.04, 0.04] |
| Parental alcohol use | -0.02 | 0.02 | [-0.07, 0.03] |
|  |  |  |  |
| *Fear of negative social evaluation* |  |  |  |
| LGB (Heterosexual=ref.) | 0.17 | 0.09 | [-0.01, 0.35] |
| Boy (girl=ref.) | -0.19 | 0.05 *^**^* | [-0.29, -0.10] |
| Age wave 4 | -0.12 | 0.04 *^*^* | [-0.20, -0.03] |
| Parental SES | 0.20 | 0.04 *^**^* | [0.13, 0.27] |
| Ethnic minority (majority=ref.) | -0.12 | 0.09 | [-0.30, 0.07] |
| Childhood events | -0.03 | 0.03 | [-0.09, 0.04] |
| Parental internalizing problems | 0.05 | 0.03 | [-0.01, 0.11] |
| Perinatal problems | 0.05 | 0.02*^*^* | [0.01, 0.09] |
| Long-term difficulties | -0.02 | 0.03 | [-0.09, 0.04] |
| Early life stress | 0.02 | 0.01 | [-0.01, 0.05] |
| Parental smoking | -0.05 | 0.02 ^*^ | [-0.09, -0.01] |
| Parental alcohol use | 0.02 | 0.02 | [-0.03, 0.06] |
| Peer victimization | 0.08 | 0.03 *^**^* | [0.02, 0.14] |
| Parental guilt inducing | 0.04 | 0.03 | [-0.02, 0.10] |
| Parental angry outbursts | 0.10 | 0.03 *^**^* | [0.04, 0.16] |
| Parental rejection | 0.04 | 0.03 | [-0.01, 0.10] |
|  |  |  |  |
| *Lack of social support* |  |  |  |
| LGB (Heterosexual=ref.) | -0.05 | 0.10 | [-0.25, 0.15] |
| Boy (girl=ref.) | 0.43 | 0.06 *^**^* | [0.33, 0.54] |
| Age wave 4 | 0.06 | 0.05 | [-0.04, 0.15] |
| Parental SES | -0.06 | 0.04 | [-0.13, 0.02] |
| Ethnic minority (majority=ref.) | -0.17 | 0.10 | [-0.37, 0.03] |
| Childhood events | -0.06 | 0.04 | [-0.13, 0.01] |
| Parental internalizing problems | 0.04 | 0.04 | [-0.03, 0.11] |
| Perinatal problems | -0.04 | 0.02 | [-0.09, 0.00] |
| Long-term difficulties | 0.01 | 0.04 | [-0.06, 0.08] |
| Early life stress | -0.01 | 0.02 | [-0.02, 0.04] |
| Parental smoking | 0.00 | 0.02 | [-0.05, 0.04] |
| Parental alcohol use | -0.03 | 0.02 | [-0.08, 0.01] |
| Peer victimization | 0.13 | 0.03 ^**^ | [0.07, 0.20] |
| Parental guilt inducing | 0.01 | 0.03 | [-0.05, 0.08] |
| Parental angry outbursts | 0.01 | 0.03 | [-0.06, 0.07] |
|  |  |  |  |
| *Substance use norms peers* |  |  |  |
| LGB (Heterosexual=ref.) | 0.05 | 0.09 | [-0.13, 0.23] |
| Boy (girl=ref.) | 0.11 | 0.05 ^*^ | [0.01, 0.21] |
| Age wave 4 | 0.28 | 0.04 *^**^* | [0.19, 0.36] |
| Parental SES | -0.08 | 0.04 *^*^* | [-0.15, 0.00] |
| Ethnic minority (majority=ref.) | 0.08 | 0.10 | [-0.11, 0.26] |
| Childhood events | 0.08 | 0.03 *^*^* | [0.01, 0.14] |
| Perinatal problems | -0.04 | 0.02 *^*^* | [-0.09, 0.00] |
| Long-term difficulties | -0.01 | 0.03 | [-0.07, 0.06] |
| Parental internalizing problems | -0.02 | 0.03 | [-0.09, 0.04] |
| Early life stress | -0.02 | 0.01 | [-0.05, 0.00] |
| Parental smoking | 0.10 | 0.02 ^**^ | [0.06, 0.14] |
| Parental alcohol use | 0.07 | 0.02 ^**^ | [0.02, 0.11] |
| Peer victimization | -0.05 | 0.03 | [-0.11, 0.01] |
| Parental guilt inducing | 0.13 | 0.03*^**^* | [0.07, 0.18] |
| Parental angry outbursts | 0.06 | 0.03 | [0.00, 0.11] |
|  |  |  |  |
| *Internalizing problems* |  |  |  |
| LGB (Heterosexual=ref.) | 0.22 | 0.09 *^*^* | [0.05, 0.39] |
| Boy (girl=ref.) | -0.30 | 0.04 *^**^* | [-0.38, -0.22] |
| Age wave 4 | 0.06 | 0.04 | [-0.02, 0.13] |
| Parental SES | -0.07 | 0.03 ^*^ | [-0.13, -0.01] |
| Ethnic minority (majority=ref.) | 0.15 | 0.08 | [-0.02, 0.31] |
| Childhood events | 0.07 | 0.03 *^*^* | [0.01, 0.13] |
| Parental internalizing problems | 0.06 | 0.03 ^*^ | [0.01, 0.12] |
| Perinatal problems | -0.04 | 0.02 ^*^ | [-0.08, -0.01] |
| Long-term difficulties | 0.11 | 0.03 *^**^* | [0.05, 0.18] |
| Early life stress | 0.01 | 0.01 | [-0.02, 0.03] |
| Parental smoking | 0.01 | 0.02 | [-0.03, 0.04] |
| Parental alcohol use | 0.01 | 0.02 | [-0.02, 0.05] |
| Peer victimization | 0.13 | 0.03 *^**^* | [0.08, 0.18] |
| Parental guilt inducing | 0.05 | 0.03 | [-0.01, 0.11] |
| Parental angry outbursts | 0.08 | 0.02 *^**^* | [0.03, 0.13] |
| Parental rejection | 0.21 | 0.02 *^**^* | [0.16, 0.25] |
| Fear of negative social evaluation | 0.35 | 0.02 *^**^* | [0.31, 0.39] |
| Lack of social support | 0.08 | 0.02 *^**^* | [0.03, 0.12] |
|  |  |  |  |
| *Smoking* |  |  |  |
| LGB (Heterosexual=ref.) | 0.37 | 0.12 *^**^* | [0.14, 0.61] |
| Boy (girl=ref.) | -0.05 | 0.07 | [-0.19, 0.10] |
| Age wave 4 | 0.11 | 0.06 | [-0.01, 0.23] |
| Parental SES | -0.14 | 0.05 *^**^* | [-0.24, -0.04] |
| Ethnic minority (majority=ref.) | -0.15 | 0.14 | [-0.36, 0.16] |
| Childhood events | 0.03 | 0.05 | [-0.06, 0.12] |
| Parental internalizing problems | 0.03 | 0.05 | [-0.07, 0.12] |
| Perinatal problems | -0.01 | 0.03 | [-0.07, 0.05] |
| Long-term difficulties | 0.05 | 0.05 | [-0.04, 0.14] |
| Early life stress | 0.02 | 0.02 | [-0.02, 0.05] |
| Parental smoking | 0.05 | 0.03 | [-0.01, 0.11] |
| Parental alcohol use | 0.05 | 0.03 | [-0.01, 0.11] |
| Peer victimization | -0.04 | 0.05 | [-0.13, 0.05] |
| Parental guilt inducing | 0.06 | 0.04 | [-0.02, 0.14] |
| Parental angry outbursts | -0.06 | 0.04 | [-0.15, 0.02] |
| Parental rejection | 0.19 | 0.04 *^**^* | [0.11, 0.27] |
| Fear of negative social evaluation | -0.15 | 0.04 *^**^* | [-0.22, -0.08] |
| Lack of social support | -0.09 | 0.04 *^*^* | [-0.17, -0.01] |
| Substance use norms peers | 0.53 | 0.04 *^**^* | [0.45, 0.61] |
|  |  |  |  |
| *Marijuana use* |  |  |  |
| LGB (Heterosexual=ref.) | 0.37 | 0.12 *^**^* | [0.13, 0.61] |
| Boy (girl=ref.) | 0.44 | 0.07 *^**^* | [0.30, 0.59] |
| Age wave 4 | 0.04 | 0.06 | [-0.07, 0.16] |
| Parental SES | 0.16 | 0.05 *^**^* | [0.06, 0.26] |
| Ethnic minority (majority=ref.) | 0.20 | 0.13 | [-0.06, 0.46] |
| Childhood events | 0.03 | 0.05 | [-0.06, 0.12] |
| Parental internalizing problems | 0.06 | 0.05 | [-0.03, 0.15] |
| Perinatal problems | -0.04 | 0.03 | [-0.10, 0.02] |
| Long-term difficulties | 0.00 | 0.05 | [-0.09, 0.10] |
| Early life stress | 0.02 | 0.02 | [-0.02, 0.05] |
| Parental smoking | 0.05 | 0.03 | [-0.01, 0.10] |
| Parental alcohol use | 0.09 | 003 ^**^ | [0.03, 0.15] |
| Peer victimization | -0.09 | 0.05 | [-0.18, 0.00] |
| Parental guilt inducing | 0.00 | 0.04 | [-0.08, 0.08] |
| Parental angry outbursts | 0.09 | 0.04 *^*^* | [0.00, 0.17] |
| Parental rejection | 0.06 | 0.04 | [-0.02, 0.14] |
| Fear of negative social evaluation | 0.00 | 0.04 | [-0.07, 0.07] |
| Lack of social support | 0.00 | 0.04 | [-0.06, 0.08] |
| Substance use norms peers | 0.36 | 0.04 *^**^* | [0.28, 0.44] |
|  |  |  |  |
| *Alcohol use* |  |  |  |
| LGB (Heterosexual=ref.) | 0.00 | 0.08 | [-0.16, 0.17] |
| Boy (girl=ref.) | 0.34 | 0.05 *^**^* | [0.24, 0.45] |
| Age wave 4 | 0.08 | 0.04 | [0.00, 0.16] |
| Parental SES | 0.13 | 0.03 *^**^* | [0.06, 0.19] |
| Ethnic minority (majority=ref.) | -0.17 | 0.09 | [-0.35, 0.01] |
| Childhood events | 0.06 | 0.03 | [-0.01, 0.12] |
| Parental internalizing problems | -0.07 | 0.03 ^*^ | [-0.13, -0.01] |
| Perinatal problems | 0.00 | 0.02 | [-0.03, 0.04] |
| Long-term difficulties | -0.01 | 0.03 | [-0.07, 0.05] |
| Early life stress | -0.01 | 0.01 | [-0.04, 0.02] |
| Parental smoking | -0.03 | 0.02 | [-0.07, 0.01] |
| Parental alcohol use | 0.09 | 0.02 ^**^ | [0.04, 0.13] |
| Peer victimization | -0.08 | 0.03 ^*^ | [-0.14, -0.01] |
| Parental guilt inducing | -0.03 | 0.03 | [-0.08, 0.02] |
| Parental angry outbursts | -0.02 | 0.03 | [-0.08, 0.04] |
| Parental rejection | 0.07 | 0.03 *^*^* | [0.02, 0.12] |
| Fear of negative social evaluation | 0.01 | 0.02 | [-0.04, 0.05] |
| Lack of social support | -0.03 | 0.03 | [-0.09, 0.03] |
| Substance use norms peers | 0.20 | 0.03 *^**^* | [0.14, 0.25] |
|  |  |  |  |
| *Peer victimization covariance with* |  |  |  |
| Parental guilt inducing | 0.06 | 0.03 | [0.00, 0.13] |
| Parental angry outbursts | 0.06 | 0.03 | [-0.01, 0.12] |
| Parental rejection | 0.13 | 0.03 *^**^* | [0.07, 0.19] |
|  |  |  |  |
| *Parental guilt inducing covariance with* |  |  |  |
| Parental angry outbursts | 0.39 | 0.03 *^**^* | [0.33, 0.45] |
| Parental rejection | 0.24 | 0.03 *^**^* | [0.17, 0.30] |
|  |  |  |  |
| *Parental angry outbursts covariance with* |  |  |  |
| Parental rejection | 0.31 | 0.03 *^**^* | [0.26, 0.37] |
|  |  |  |  |
| *Fear of negative social evaluation covariance with* |  |  |  |
| Lack of social support | 0.02 | 0.03 | [-0.03, 0.07] |
| Substance use norms peers | -0.03 | 0.02 | [-0.08, 0.02] |
|  |  |  |  |
| *Lack of social support covariance with* |  |  |  |
| Substance use norms peers | -0.10 | 0.03 *^**^* | [-0.15, -0.05] |
|  |  |  |  |
| *Internalizing problems covariance with* |  |  |  |
| Smoking | 0.07 | 0.03 *^*^* | [0.01, 0.12] |
| Marijuana use | 0.07 | 0.03 *^**^* | [0.02, 0.13] |
| Alcohol use | -0.01 | 0.02 | [-0.04, 0.03] |
|  |  |  |  |
| *Smoking covariance with* |  |  |  |
| Marijuana use | 0.51 | 0.04 *^**^* | [0.44, 0.59] |
| Alcohol use | 0.21 | 0.04 *^**^* | [0.14, 0.25] |
|  |  |  |  |
| *Alcohol use covariance with* |  |  |  |
| Marijuana use | 0.15 | 0.03 *^**^* | [0.09, 0.21] |
|  |  |  |  |
| *Intercepts* |  |  |  |
| Parental guilt inducing | 0.00 | 0.04 | [-0.07, 0.07] |
| Parental angry outbursts | 0.12 | 0.04 ^**^ | [0.05, 0.20] |
| Parental rejection | -0.01 | 0.03 | [-0.08, 0.06] |
| Fear of negative social evaluation | 0.08 | 0.03 *^*^* | [0.01, 0.15] |
| Lack of social support | -0.19 | 0.04 ^**^ | [-0.26, -0.12] |
| Substance use norms peers | -0.06 | 0.00 *^*^* | [-0.12, 0.01] |
| Internalizing problems | 0.10 | 0.03 ^**^ | [0.04, 0.16] |
| Alcohol use | -0.14 | 0.03 *^**^* | [-0.20, 0.08] |
|  |  |  |  |
| *Thresholds* |  |  |  |
| Peer victimization$1 | 0.36 | 0.05 ^**^ | [0.27, 0.45] |
| Smoking $1 | 0.13 | 0.05 *^**^* | [0.03, 0.23] |
| Marijuana use$1 | 0.69 | 0.05 ^**^ | [0.59, 0.80] |
|  |  |  |  |
| *Residual variances* |  |  |  |
| Parental guilt inducing | 0.98 | 0.07 *^**^* | [0.85, 1.12] |
| Parental angry outbursts | 0.95 | 0.03 *^**^* | [0.89, 1.01] |
| Parental rejection | 0.94 | 0.04 *^**^* | [0.86, 1.03] |
| Fear of negative social evaluation | 0.91 | 0.03 *^**^* | [0.85, 0.97] |
| Lack of social support | 0.90 | 0.03 *^**^* | [0.86, 0.97] |
| Substance use norms peers | 0.91 | 0.03 *^**^* | [0.84, 0.95] |
| Internalizing problems | 0.64 | 0.03 *^**^* | [0.58, 0.69] |
| Alcohol use | 0.87 | 0.06 *^**^* | [0.76, 0.98] |
| Note: ^*^ *p* < 0.05, ^**^ *p* < 0.01, two-sided | | |  |
