# Supplementary material for: Explaining Health Disparities between Heterosexual and LGB Adolescents by Integrating the Minority Stress and Psychological Mediation Frameworks: Findings from the TRAILS Study
Source: J Youth Adolesc. 2020 Feb 19;49(9):1767–82. doi: 10.1007/s10964-020-01206-0 (PMC7423798; doi:10.1007/s10964-020-01206-0)

**Appendix B. Results lagged dependent variable models**

Variable operationalizations and model specification decisions differed in some respects in models including lagged dependent variables compared to the main results, as both models tried to fulfill slightly different aims. In the main models, between-person differences in mediators and health outcomes were approximated, whereas in models including lagged dependent variables the aim was to estimates effects of minority stress and psychological mediators on change in health outcomes over time.

Toward this aim, peer victimization and smoking were recoded in order to reflect current behaviors, instead of current and previous behavior in our main models. Peer victimization was operationalized using wave 3 information (none versus any wave 3 victimization). Smoking was recoded to reflect current smoking behavior (*I smoke every now and then, but not every day* or *I smoke every day*).

In terms of model specification, concurrent instead of lagged effects of mediators on change in dependent variables were estimated. Lags between waves were 2 to 3 years, whereas mediators were expected to induce change in health outcomes within a matter of weeks or months. Therefore, concurrent effects estimates are likely more accurate than lagged effects estimates (Vaisey & Miles, 2017). This resulted in estimating four models: One minority stress model estimating effects of minority stressors measured at wave 3 on wave 3 health outcomes controlling for wave 2 health outcomes; One minority stress model estimating effects of minority stressors measured at wave 4 on wave 4 health outcomes controlling for wave 3 health outcomes; One psychological mediation model estimating effects of mediators measured at wave 3 on wave 3 health outcomes controlling for wave 2 health outcomes; and one psychological mediation model estimating effects of minority stressors measured at wave 4 on wave 4 health outcomes controlling for wave 3 health outcomes. Summaries of these models are displayed in Figures B1 to B4 (full path coefficients available upon request). Although statistical significance with regard to some of the direct paths were different from those presented in the main text (see Figures), overall conclusions remain unchanged: Controlling for multiple testing, statistically significant evidence was found for the association between sexual orientation and internalizing problems being mediated by peer victimization (*b* (*se*) *=* .20 (.05), *95%* *CI* [.10, .31]) and parental rejection (*b* (*se*) *=* .06 (.02), *95%* *CI* [.02, .10]).

**References**

Vaisey, S., & Miles, A. (2017). What you can—and can’t—do with three-wave panel data. *Sociological Methods and Research*, *46*(1), 44–67. doi: 10.1177/0049124114547769


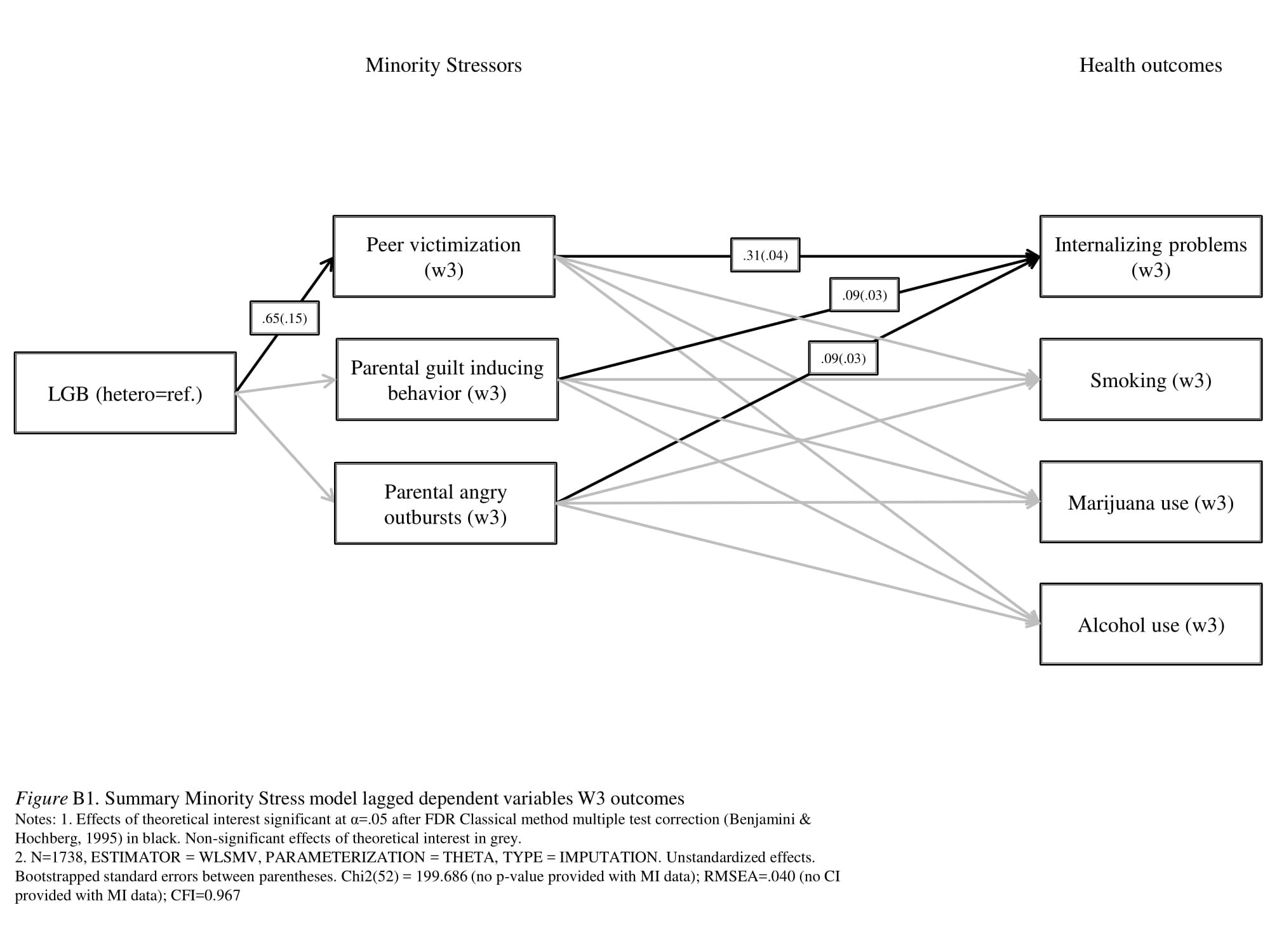


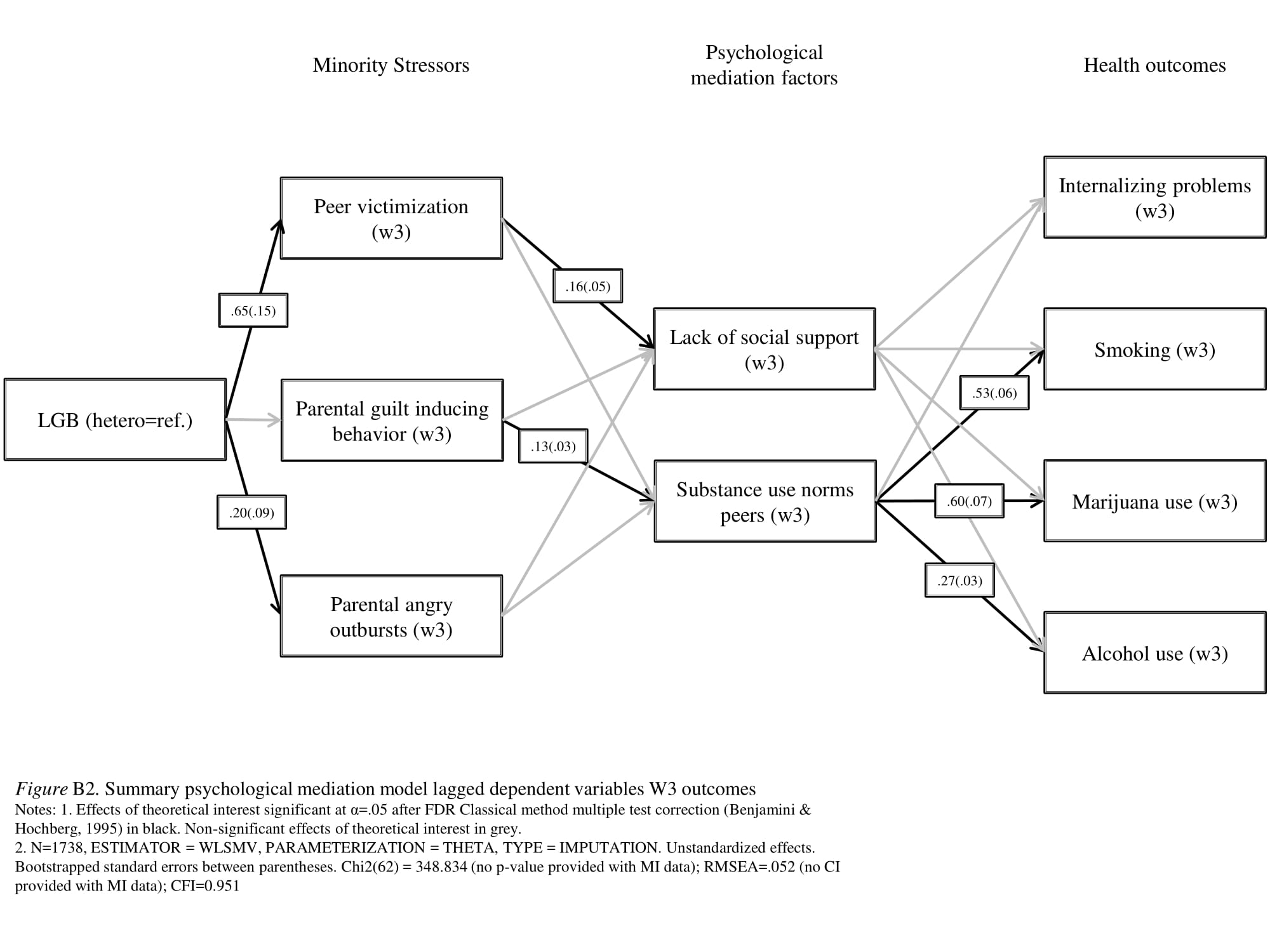


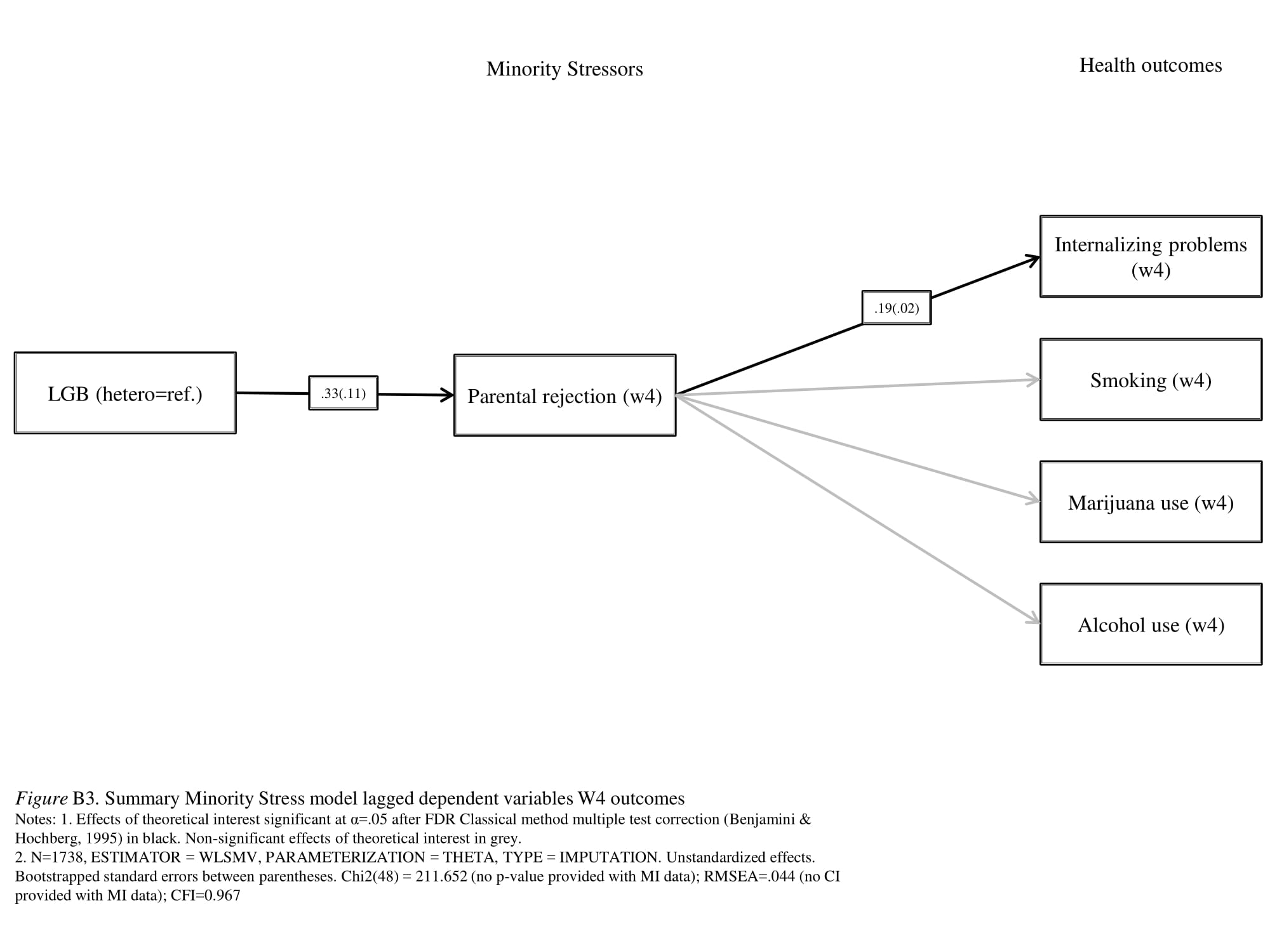


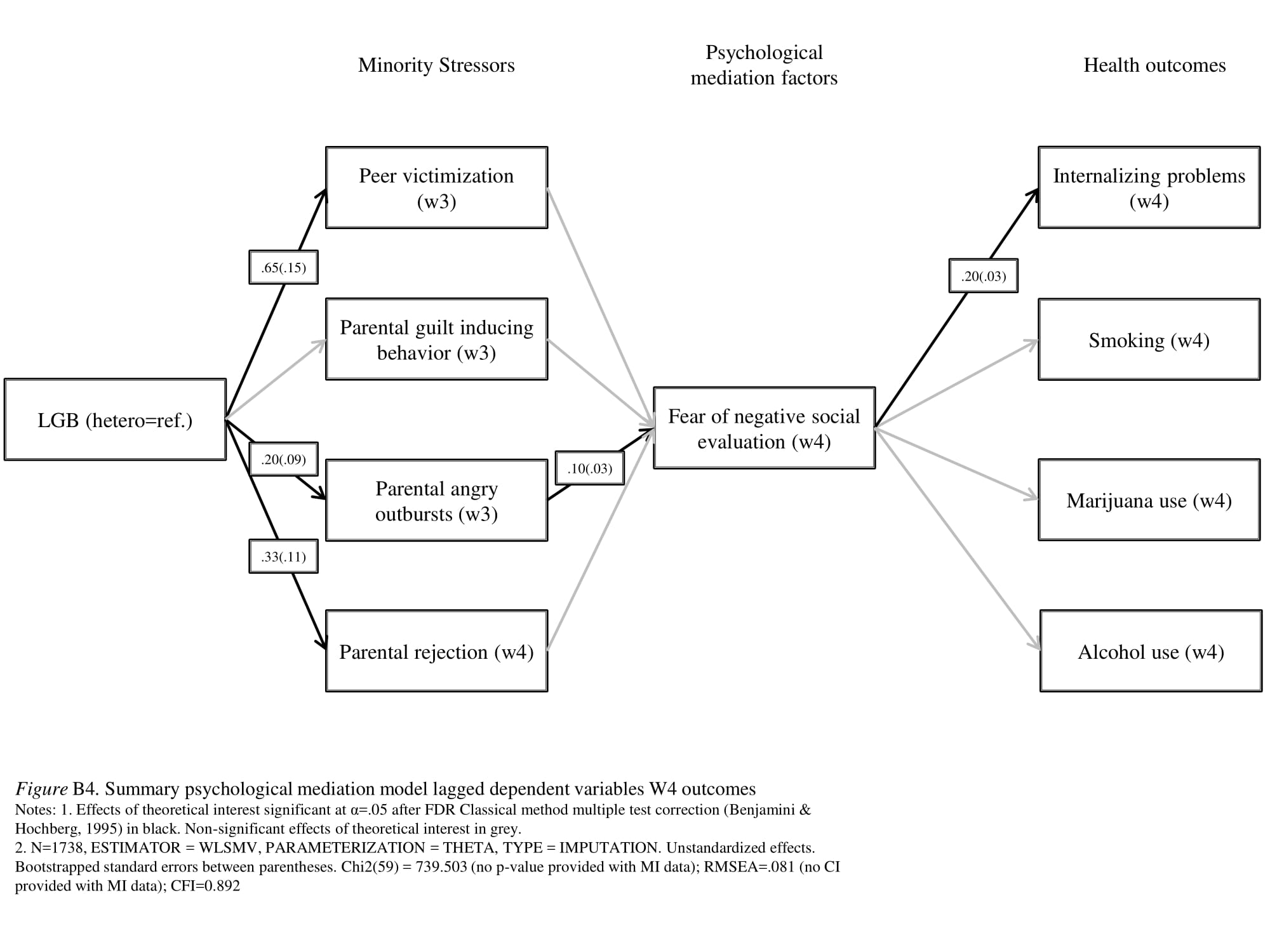

Supplement: Supplementary file 2 — Appendix B [file 10964_2020_1206_MOESM2_ESM.docx]
